# Immunotherapy in non-small cell lung cancer: Past, present, and future directions

**DOI:** 10.3389/fonc.2022.877594

**Published:** 2022-08-02

**Authors:** Salman R. Punekar, Elaine Shum, Cassandra Mia Grello, Sally C. Lau, Vamsidhar Velcheti

**Affiliations:** Perlmutter Cancer Center, New York University (NYU) Langone Health, New York, NY, United States

**Keywords:** Immunotherapy, immune checkpoint blockade, PD-1, PD-L1, CTLA-4, biomarkers, NSCLC

## Abstract

Many decades in the making, immunotherapy has demonstrated its ability to produce durable responses in several cancer types. In the last decade, immunotherapy has shown itself to be a viable therapeutic approach for non-small cell lung cancer (NSCLC). Several clinical trials have established the efficacy of immune checkpoint blockade (ICB), particularly in the form of anti-programmed death 1 (PD-1) antibodies, anti-cytotoxic T-lymphocyte-associated protein 4 (CTLA-4) antibodies and anti-programmed death 1 ligand (PD-L1) antibodies. Many trials have shown progression free survival (PFS) and overall survival (OS) benefit with either ICB alone or in combination with chemotherapy when compared to chemotherapy alone. The identification of biomarkers to predict response to immunotherapy continues to be evaluated. The future of immunotherapy in lung cancer continues to hold promise with the development of combination therapies, cytokine modulating therapies and cellular therapies. Lastly, we expect that innovative advances in technology, such as artificial intelligence (AI) and machine learning, will begin to play a role in the future care of patients with lung cancer.

## Introduction

Immune modulation has long been studied as a potential therapeutic target in the battle against disease. In 1798, Edward Jenner coined the term “vaccination” as he popularized and spread the concept of inoculation with cowpox to prevent smallpox across Europe (1). Almost a hundred years later, across the Atlantic, William Coley began injecting bacterial products known as Coley’s Toxins into patients with sarcomas (2). This was perhaps modern medicine’s first foray into immuno-oncology.

In 1909, Paul Ehrlich proposed the hypothesis that the aberrant transformation of cells was an inherent and common process, but host defense mechanisms prevent them from turning into cancer (3). Paul Ehrlich’s hypothesis was further refined in the 1950s and 1960s by Lewis Thomas and Frank Burnet, who hypothesized that an antigenic response by the immune system against tumor cells formed the basis of the cancer immunosurveillance hypothesis (4). Around the turn of the century, this hypothesis was further molded into the cancer immunoediting hypothesis, largely known by its three “E”s: elimination, equilibrium, and escape (5). Normal cells undergo malignant transformation due to any number of factors, which induces the expression of tumor-associated antigens that promote immune-related *elimination* of malignancy cells, classically referred to as immunosurveillance. In a subset of these situations, the immune system will not be able to completely eradicate a malignant group of cells and these cells will exist in *equilibrium*. Lastly, some of these cells will undergo immune *escape* to ultimately develop into clinically significant cancers (4–7).

The discovery and description of the programmed death (PD) pathway as mediators of tumor immune evasion changed the trajectory of immuno-oncology (8–12). The process of T cell activation begins when the T cell receptor engages with antigen presented by a major histocompatibility protein (MHC). However, T cell activation does not occur unless a second co-stimulatory signal is received. On the other hand, inhibitory checkpoints, such as CTLA-4 and PD-1, lead to T cell anergy or apoptosis when engaged. In normal states, these checkpoints are crucial in controlling immune activation and preventing collateral tissue damage from autoimmunity. In states of chronic inflammation or cancer, exhausted T cells are found to upregulate expression of inhibitory CTLA-4 and PD-1 molecules. Monoclonal antibodies against CTLA-4, PD-1/PD-L1 are designed to reinvigorate exhausted T cells and restore immune-mediated elimination of malignant cells, a strategy that has highly successful against multiple tumor subtypes including NSCLC (13–19).

The 2018 Nobel Prize in Physiology or Medicine was awarded to Drs. James Allison and Tasuku Honjo for their work in describing the cytotoxic T-lymphocyte-associated antigen 4 (CTLA-4) and PD-1 pathways as checkpoints and further showing the inhibition of these checkpoints improve T-cell mediated anti-tumor effects (20). Ipilimumab, an anti-CTLA-4 antibody, gained widespread attention when it was shown to improve survival in advanced melanoma (21). Nivolumab, the first monoclonal antibody (mAb) against PD-1, first demonstrated its utility in the treatment of metastatic melanoma, non-small cell lung cancer (NSCLC), and advanced renal cell carcinoma (RCC) and further showed survival benefit in a variety of malignancies such as head and neck squamous cell carcinoma (HNSCC) amongst others (22–27). Another well-known PD-1 mAb, pembrolizumab, generated similar excitement. Pembrolizumab has shown positive activity in many cancers, including melanoma, NSCLC, triple negative breast cancer (TNBC), and HNSCC (28–34). Aside from nivolumab and pembrolizumab, many other PD-1 and PD-L1 modulators have been developed and have shown activity in small cell lung cancer (SCLC), cutaneous squamous cell carcinoma, bladder cancer, and TNBC (35–42).

## The current immunotherapy landscape for the treatment of NSCLC

### Immunotherapy in early stage NSCLC

#### Adjuvant immunotherapy in resected NSCLC

Until recently, chemotherapy with a platinum doublet has been the mainstay of adjuvant therapy after curative resection of early stage NSCLC. The IMpower010 study is the first clinical trial to demonstrate meaningful improvements in disease free survival (DFS) with immunotherapy in stage IB-IIIA NSCLC. Patients enrolled in this study recent standard of care chemotherapy were randomized to adjuvant atezolizumab, a PD-L1 inhibitor, for one year or best supportive care. At interim analysis, patients with PD-L1 positive (≥1%), stage II-IIIA disease who received atezolizumab had a superior median DFS compared to best supportive care (stratified HR 0.66, 95%CI 0.50-0.88, p=0.004) (43). Although the data on overall survival is still immature at this point, the improvements in DFS is felt to be clinically meaningful. It is important to note that immunotherapy is an addition and not a replacement for adjuvant chemotherapy. In addition, patients with resected EGFR mutant NSCLC should receive an adjuvant tyrosine kinase inhibitor (TKI) (44) rather than immunotherapy. Although there is no direct comparison, the effectiveness of immunotherapy in EGFR mutant NSCLC is low in advanced disease based on pooled analysis of several trials including KEYNOTE-010 and CheckMate 057 (45–48) and the use of adjuvant EGFR TKIs demonstrated impressive improvements in DFS in the phase 3 ADAURA trial of 682 patients with stage IB to IIIA EGFR mutant NSCLC. This trial showed a 2 year DFS of 89% vs 53% patients treated with osimertinib compared to those treated with placebo (44). Based on interim results of the IMpower010 study, atezolizumab received approval from the US Food and Drug Administration (FDA) for use as adjuvant therapy in resected stage II-IIIA NSCLC with PD-L1≥1%. It is the first study to demonstrate that immunotherapy can improve the cure rates of patients with early stage lung cancer. The phase 3 KEYNOTE-091 trial further supported the use of immunotherapy in the adjuvant study. This study, presented at ESMO in 2022, evaluated the use of pembrolizumab versus placebo in stage IB to IIIA NSCLC after resection and adjuvant chemotherapy (if indicated) and found improved median DFS (53.6 months vs 42.0 months; HR 0.76; 95% CI 0.63-0.91; p<0.01) in patients receiving pembrolizumab. It is important to note that pembrolizumab was well tolerated in this trial, with a median number of doses of 17 in the investigational arm compared to 18 in the placebo arm. Not surprisingly, patients who received pembrolizumab experienced more grade ≥3 adverse events than those who did not (34.1% vs 25.8%). There was also a treatment discontinuation rate of 19.8% in the pembrolizumab arm compared to 5.9% in the placebo arm (49), suggesting that the DFS benefit must be considered in context of the relatively favorable safety and tolerability profile of this agent.

#### Neoadjuvant immunotherapy in NSCLC

Immunotherapy has also been explored in the neoadjuvant setting. Neoadjuvant therapy has several potential benefits: 1) downstage tumor prior to definitive surgery, 2) early treatment of micrometastatic disease, 3) improved T cell priming from neoantigens derived from the primary tumor and 4) provide prognostic information based on tumor pathological response to treatment.

Forde et al., published the first pilot study investigating two preoperative doses of nivolumab, a PD-1 inhibitor, in patients with stage I, II, or IIIA NSCLC in the CheckMate-159 trial (50). Although the number of patients was small (n=21), they reported a major pathological response (MPR) in 9 of 20 resected tumors (45%). They also showed that this treatment was associated with few side effects and no delays to surgery. The Lung Cancer Mutation Consortium (LCMC) 3 trial is the largest study investigating the use of single agent ICI as neoadjuvant therapy in patients with resectable stage IB-IIIB NSCLC. A total of 181 patients were enrolled. Patients were treated with 2 cycles of neoadjuvant atezolizumab followed by surgery, then completion of adjuvant atezolizumab for one year. The MPR rate, defined as <10% viable tumor cells, was 21%, with a 7% complete pathologic response rate, supporting the findings from the smaller studies. Notably, in 10% of patients, surgery occurred outside of the protocol window and 11% of patients ultimately did not undergo surgery (51). Encouragingly, the exploratory analyses on DFS and OS, stratified by stage, were similar to historical controls.

PD-1/PD-L1 inhibitors in combination with CTLA-4 inhibitors or chemotherapy have also been investigated as neoadjuvant therapy. The phase 2 NEOSTAR trial evaluated MPR in patients receiving neoadjuvant nivolumab alone or nivolumab and ipilimumab. Of the 44 patients enrolled, there was a 24% MPR rate in the nivolumab arm and 50% in the nivolumab and ipilimumab arm. 22% of patient had surgery after 42 days of completion of neoadjuvant treatment (52). In a single-arm phase 2 trial evaluating combination chemotherapy with atezolizumab in resectable NSCLC, 57% of patients achieved major pathologic response (53). The NADIM trial evaluated the role of neoadjuvant nivolumab in combination with platinum doublet chemotherapy in resectable stage IIIA NSCLC (54). In this trial, patients were treated with paclitaxel and carboplatin plus nivolumab for 3 cycles prior to surgery followed by 1 year of adjuvant nivolumab. Progression-free survival was found to be 77.1% at 24 months.

The CheckMate-816 is the only phase 3 trial in this space investigating neoadjuvant chemotherapy with or without nivolumab in patients with resectable stage IB-IIIA NSCLC. The primary endpoint of pathological complete response (pCR) was significant higher in patients who received nivolumab and chemotherapy compared to chemotherapy alone (24% vs 2.2%, OR 13.94, 99% CI, 3.49-55.75, p<0.0001). Benefit was seen in both PD-L1 <1% and PD-L1≥1% subgroups (55). MPR rates were 36.9% in the nivolumab-containing group and 8.9% in patients receiving chemotherapy alone. There was no increase in grades 3 or 4 drug-related adverse events and no safety signals on surgical complications or outcomes (56).

Neoadjuvant immunotherapy has strong mechanistic rationale to improve anticancer T cell mediated immunity. This can be due to expansion of tumor infiltrating lymphocytes *via* PD-L1 and PD-L2 expressing dendritic cells. Furthermore, these dendritic cells (after treatment with PD-(L)1 blockade) can better present tumor associated antigens in regional lymph nodes, further enhancing antitumor immunity (57).

As of March 2022, neoadjuvant immunotherapy (nivolumab) was approved by the FDA in combination with platinum-doublet chemotherapy based on the CheckMate-816 trial. Improved Event Free Survival (EFS) was recently reported in this trial – 31.6 months with nivolumab plus chemotherapy versus 20.8 months with chemotherapy alone (HR for progression, recurrence or death: 0.63, 97.38% CI: 0.43-0.91, p=0.005) (58). While the above-mentioned trials demonstrate benefit, it is important to await data maturity to ensure that benefits in pathologic response translates to similar benefits in survival. Additionally, it is important to exercise caution given that an important limitation of neoadjuvant therapy stems from the inherent delay to definitive surgical resection that neoadjuvant treatment creates for patients. To this end, investigators have proposed the integration of surgical endpoints and metrics to help further clarify this issue (56). Lastly, just as in the metastatic setting, complete genetic profiling of early stage tumors is critical prior to initiation of therapy, as recurrence of tumors harboring driver mutations have been described (59).

### Immunotherapy in locally advanced NSCLC

The PACIFIC trial is a randomized phase 3 trial of 713 patients with unresectable stage III NSCLC who were treated with concurrent chemoradiotherapy, followed by durvalumab, a PD-L1 inhibitor, consolidation versus placebo. The trial showed PFS benefit in patients treated with durvalumab as compared to placebo (PFS 16.8 months vs 5.6 months, HR 0.52, 95% CI 4.6-7.8). In addition, an OS benefit was demonstrated with durvalumab (HR 0.68; 99.73% CI, 0.47-0.997; p=0.0025) with a 24-month OS rate of 66.3% compared to 55.6% in the placebo group (36). An updated 5-year data analysis showed persistent benefit with a 5-year OS rate of 42.9% in the group that received durvalumab compared to 33.4% in the placebo group (HR 0.72, 95% CI 0.59-0.89), supporting the use of durvalumab after CRT as standard of care in this setting (60). The rates of severe adverse events were similar. In addition, quality of life, assessed prospectively by patient reported outcomes, was similar even with an additional year of active therapy (61).

Building upon the success of the PACIFIC trial. Investigators have also evaluated the efficacy immunotherapy combinations. The KEYNOTE-799 study is a phase II trial that investigated chemo-immunotherapy with pembrolizumab, a PD-1 inhibitor, concurrently with radiation (62). The results demonstrated a promising response rate of approximately 70% although survival data remained immature. The use of pembrolizumab concurrently with radiation did not appear to affect the rates of severe pneumonitis (~8%). The PACIFIC-2 trial moves the use of durvalumab earlier in the treatment course of patients with stage III NSCLC and is attempting to answer whether concurrent chemoradiation and immunotherapy is superior to chemoradiation with consolidative immunotherapy (63). Given that many patients are not candidates for concurrent chemoradiation, the phase 2 PACIFIC-6 trial is assessing the safety of durvalulmab after sequential chemoradiation. Data presented in 2022 showed similar safety to that seen in the PACIFIC trial suggesting that this may also represent a reasonable treatment strategy in patients unable to undergo concurrent chemoradiation (64). In the phase II COAST study, durvalumab was combined with olecumab (anti-CD73 mAb) or monalizumab (anti-NKG2A mAb) as consolidative treatment of unresectable stage III NSCLC after concurrent chemoradiation. Both the durvalumab plus olecumab and the durvalumab plus monalizumab arms showed improved ORR compared to durvalumab alone (38.3%, 37.1%, and 25.5% respectively) as well as improved median PFS (not reached, 15.1 months, and 6.3 months, respectively). Toxicity was similar across all three arms, suggesting that combinatorial approaches to consolidation treatment can improve outcomes (65).

### Immunotherapy in advanced NSCLC

There are now several FDA approved regimens for the treatment of metastatic NSCLC, giving patients and physicians options to individualize therapy given specific characteristics. There are options to avoid chemotherapy when appropriate. The approved first line immunotherapy regimens and their efficacy data are summarized in **Table 1**.

**Table 1:** First-line FDA approved treatment options for EGFR/ALK wild type, metastatic NSCLC.

Registration Study	Regimen	Patient Selection	ORR	PFS	OS
**PD-1/PD-L1 Monotherapy**					
KEYNOTE-024 Reck et al. (33)	Pembrolizumab	PD-L1 ≥50%	45%	10.3 months	26.3 months
IMpower110 Spigel et al. (66, 67)	Atezolizumab	PD-L1 ≥50% (TC3) or IC ≥10% (IC3)	38%	8.1 months	20.2 months
EMPOWER-Lung 1 Sezer et al. (68)	Cemiplimab	PD-L1 ≥50%	39%	8.2 months	NR
KEYNOTE-042 Mok et al. (69)	Pembrolizumab	PD-L1 ≥1%	27%	5.4 months	16.7 months
**PD-1/PD-L1 Inhibitors and Chemotherapy**					
KEYNOTE-189 Gandhi et al. (32)	Pembrolizumab + chemotherapy	All PD-L1 Non-squamous	48%	8.8 months	22.0 months
KEYNOTE-407 Paz-Ares et al. (70, 71)	Pembrolizumab + chemotherapy	All PD-L1 Squamous	58%	6.4 months	15.9 months
IMpower 130 West et al. (72)	Atezolizumab + chemotherapy	All PD-L1 Non-squamous	49%	7.0 months	18.6 months
**PD-1/PDL-1 and CTLA-4 Inhibitors**					
CheckMate-227 Hellmann et al. (73)	Nivolumab + Ipilimumab	PD-L1 ≥1%	36%	5.1 months	17.1 months
CheckMate-9LA Paz-Ares et al. (74, 75)	Nivolumab + Ipilimumab + chemotherapy (x 2 cycles)	All PD-L1	38%	6.8 months	14.1 months
**PD-1/PD-L1 and Angiogenesis Inhibitors**					
IMpower150 Socinski et al. (38)	Atezolizumab + Bevacizumab + chemotherapy	All PD-L1	64%	8.3 months	19.2 months

#### Checkpoint inhibitor monotherapy

##### First line treatment

CheckMate-012, published in 2016, was a multicenter phase 1 trial of 52 patients that studied first-line nivolumab in advanced NSCLC and yielded positive safety data as well as durable response rates (76). This trial effectively brought nivolumab into the front-line setting and set the stage for further trials. Following this, CheckMate-026 selected patients with stage IV or recurrent NSCLC with PD-L1 expression of ≥5% to receive first-line nivolumab or platinum-based chemotherapy in this phase 3 trial. While this trial did not show a significant PFS or survival benefit, it did show significantly fewer treatment-related grade 3 and 4 adverse events (18% of patients who received nivolumab vs 51% of patients who received chemotherapy) (77). Possible explanations for why this trial did not demonstrate PFS or OS benefits include imbalances between groups that conferred better prognoses and imbalances in patients with PD-L1 ≥ 50%.

The KEYNOTE-024 study was a phase 3 trial in which 305 patients whose tumors had a PD-L1 tumor proportion score (TPS) ≥50% and lacked *EGFR* or *ALK* alterations were randomized to single agent pembrolizumab or chemotherapy in the first-line setting. Median PFS was 10.3 months versus 6 months in the chemotherapy group (p <0.001). Additionally, ORR was higher in the pembrolizumab group (44.8% versus 27.8%) compared to the chemotherapy group as well as duration of response (not reached versus 6.3 months). Updated analysis confirmed the value of single-agent pembrolizumab in this setting (OS of 30 months versus 14.2 months with HR of 0.63 (95% CI, 0.47-0.86), and when adjusted for crossover, the HR was 0.49 (95% CI, 0.34-0.69). Serious TRAEs were lower in patients treated with pembrolizumab compared to chemotherapy (26.6% versus 53.3%) (33). This trial led to the first approval of an ICI for patients with treatment naïve metastatic NSCLC (78). A subsequent study of atezolizumab compared to chemotherapy demonstrated similar efficacy in metastatic NSCLC with PD-L1 expression ≥50% or immune cell (IC) PD-L1 ≥10% (median OS 20.2 months versus 13.1 months, HR 0.59, p=0.01) (66). Cemiplimab is the newest PD-1 inhibitor approved in this setting, demonstrating improvements in PFS (8.2 months vs 5.7 months, HR 0.54, 0.43-0.68, p<0.0001) and OS (median NR vs 14.2 months, HR 0.57, 0.42-0.77, p=0.0002) compared to chemotherapy in PD-L1≥50% disease (79). The similarities in efficacy across different trials suggest that this is a class effect of PD-1/PD-L1 inhibitors. Adverse events profiles are also similar and each drug has demonstrated improvements in quality of life (80–82).

Given initial success in the PD-L1 high population, single agent ICIs was investigated in tumors with any PD-L1 expression ≥1%, with mixed results. The CheckMate-026 study compared nivolumab to chemotherapy in NSCLC with PD-L1 expression of ≥5%, but failed to demonstrate improvements in PFS or OS (77). An updated analysis of atezolizumab also failed to demonstrate an improvement in OS over chemotherapy (67). However, the KEYNOTE-042 study, which compared pembrolizumab to chemotherapy in tumors with PD-L1 ≥1%, demonstrated statistically improved OS across all PD-L1 expression levels: TPS ≥50% (HR 0.69, 95% CI 0.56-0.85 p=0.0003), TPS ≥20% (HR 0.77, 95% CI 0.64-0.92, p=0.002), and TPS>1% (HR 0.81, 95% CI 0.71-0.93, p= 0.0018) (69). There is no obvious explanation for differences in efficacy across the different drugs, but it is clear that PD-L1 is a predictive biomarker for single agent ICIs and efficacy is generally lower among patients with low PD-L1 expression. Pembrolizumab is the only ICI approved as monotherapy in the first line setting in NSCLC with low PD-L1 expression (PD-L1≥1%).

##### Second line and beyond treatment

In patients who have not received ICIs in the first line setting, several ICIs are approved as monotherapy for subsequent treatment. Several pivotal studies demonstrated improved overall survival with nivolumab compared to docetaxel in previous treated NSCLC (45, 83–85). CheckMate-017 randomized 272 patients with progressive squamous NSCLC to either nivolumab or docetaxel and reported improved overall survival (OS) in the nivolumab arm compared to the docetaxel arm (9.2 months vs 6 months, HR 0.59, 95% CI 0.44-0.79, p< 0.001) as well as an improved ORR (20% vs 9%) (85). The CheckMate-057 trial, designed similarly to CheckMate-017 but for patients with nonsquamous histology, also randomized patients to either nivolumab or docetaxel. The trial met its primary endpoint of OS, in which the nivolumab arm was found to have improved OS compared to the docetaxel arm (12.2 months vs 9.4 months, HR 0.73, 96% CI 0.59-0.89, p = 0.002). Two year follow up showed that nivolumab continued to demonstrate OS benefit compared to docetaxel (86).

Historically, the use of pembrolizumab emerged as a key immunotherapy agent in NSCLC based on the early KEYNOTE trials. In the phase 1 KEYNOTE-001 trial, published in 2015, 495 patients with advanced NSCLC were treated with pembrolizumab. This study demonstrated the safety of pembrolizumab as well as an anti-tumor effect in this population. Furthermore, this trial showed that PD-L1 expression of >50% was associated with greater efficacy compared to PD-L1 expression of <1% and PD-L1 expression of between 1% and 49% (30).

The KEYNOTE-010 trial, published in Lancet in 2016, was a randomized phase 2/3 study in which over a thousand patients with previously treated advanced NSCLC with PD-L1 expression of at least 1% were randomized to either pembrolizumab (2mg/kg or 10mg/kg) or docetaxel. Patients treated with pembrolizumab experienced improved OS (10.4 months with pembrolizumab 2mg/kg HR 0.71, 95% CI 0.58-0.88, p=0.0008], 12.7 months with pembrolizumab 10mg/kg [HR 0.61, 95% CI 0.49-0.75, p<0.0001]) compared to docetaxel (8.5 months). PFS was not significantly different between these three groups. In patients with PD-L1 expression of ≥50%, OS was more pronounced at 14.9 or 17.3 months compared to 8.2 months with docetaxel (pembrolizumab 2mg/kg: 14.9 months, HR 0.54, 95% CI 0.38-0.77, p=0.0002; pembrolizumab 10mg/kg; 17.3 months, HR 0.50, 95% CI 0.36-0.70, p<0.0001). Furthermore, fewer severe treatment-related adverse events (TRAEs) were reported with pembrolizumab (13% with 2mg/kg and 16% with 10mg/kg) compared to docetaxel (35%) (45). Long-term follow up, at 43 months, presented in 2018 showed durable responses with pembrolizumab (87).

The OAK study comparing atezolizumab and chemotherapy demonstrated similar findings in patients with all PD-L1 expressions (median OS 13.8 vs 9.6 months) (83). In addition, durvalumab was studied in the third line and beyond setting. In these heavily pre-treated patients, there was still a statistically significant and clinically meaningful improvement in survival of patients with PD-L1≥25% (median OS 11.7 vs. 6.8 months), underscoring the importance of ICIs as a backbone therapy in NSCLC (88).

#### Checkpoint inhibitor combined with chemotherapy

The addition of chemotherapy to PD-1/PD-L1 inhibitors is thought to potentiate the anti-tumor immune response by enhancing neoantigen presentation after destruction. In clinical trials, the combination demonstrated significant activity and is a standard first line treatment option in metastatic NSCLC.

The efficacy of first-line pembrolizumab in combination with a platinum doublet followed by chemo-immunotherapy maintenance was established in the KEYNOTE-189 trial for nonsquamous metastatic NSCLC. The rate of OS at 12 months was improved in the pembrolizumab group (69.2% versus 49.4%, HR 0.49, 95% CI 0.38-0.64, p<0.001) as was median PFS (8.8 months versus 4.9 months, HR 0.52, 95% CI 0.43-0.64). Grade 3 or higher adverse events between the two groups were comparable (67.2% versus 65.8%) (32). Updated data showed continued OS benefit in the pembrolizumab group (22 months versus 10.7 months, HR 0.56, 95% CI 0.45-0.70). OS and PFS benefits were seen regardless of PD-L1 expression and presence of liver or brain metastases (89). This update further supports the role of pembrolizumab with platinum doublet-based therapy in the treatment of patients with metastatic nonsquamous NSCLC. For metastatic squamous NSCLC, the KEYNOTE-407 trial established the role of first-line pembrolizumab in combination with a platinum-based drug combined with a taxane, followed by immunotherapy maintenance. Combination pembrolizumab and chemotherapy demonstrated significant improvements in OS (17.1 months versus 11.6 months, HR 0.71 95% CI 0.58-0.88) and PFS (6.4 months versus 4.8 months, HR 0.56, 95% CI 0.45-0.70, p<0.001) (70, 71). Benefit was also seen regardless of PD-L1 expression. Rates of severe adverse events were also comparable between the two groups (69.8% versus 68.2%) (71).

The combination of cemiplimab and chemotherapy also demonstrated preliminary efficacy in a phase 3 study over chemotherapy alone across all PD-L1 levels (median OS 21.9 months versus 13 months, HR 0.71, p = 0.014) (90).

Studies of atezolizumab in combination with chemotherapy had mixed results. In the Impower130 study, atezolizumab, carboplatin and nab-paclitaxel was compared to chemotherapy in non-squamous NSCLC and demonstrated improvements in PFS (7.0 vs 5.5 months; HR 0.64; p<0.0001) and OS (18.6 vs 13.9 months; HR 0.69; p=0.03) (72). Based on these results, the combination was granted regulatory approval. However, the subsequent IMPower132 study, which included the same patient population, failed to improve OS when atezolizumab was combined with carboplatin/cisplatin and pemetrexed (7.6 months versus 5.2 months, HR 0.60, 95% CI 0.49-0.72, p<0.0001) (91). In squamous NSCLC, the Impower131 study, atezolizumab, carboplatin and nab-paclitaxel (A+CP) improved PFS (6.3 months versus 5.6 months, HR 0.71, 95% CI 0.60-0.85, p = 0.0001) but not OS (14.2 vs 13.5 months; HR 0.88; p=0.16) (92). There are several potential explanations for these discrepancies including the type of chemotherapy that was used and the complexity of study designs with co-primary endpoints. It also highlights the limitations of surrogate endpoints, such as PFS, which may not always translate into a survival advantage.

#### Dual checkpoint inhibitors

The CheckMate 227 study evaluated nivolumab plus ipilimumab, and nivolumab alone to chemotherapy in treatment naïve, PD-L1≥1%, metastatic NSCLC. In this trial, patients were randomized to either nivolumab plus ipilimumab, nivolumab, or chemotherapy. This trial was amended to add a co-primary endpoint of PFS in TMB high regardless of PD-L1 expression. The combination of nivolumab and ipilimumab demonstrated an improvement in OS compared to chemotherapy alone (17.1 vs 14.9 months, p=0.007). In a secondary analysis the benefit of nivolumab plus ipilimumab was seen even in patients with PD-L1<1%. Rates of serious adverse events were 32.8% and 36% in patients receiving nivolumab plus ipilimumab and chemotherapy, respectively (93). In an updated analysis, 4-year survival rates in patients with PD-L1≥50% were 37% (nivolumab plus ipilimumab), 26% (nivolumab alone) and 20% (chemotherapy), and 4-year survival rates in patients with PD-L1<1% were 24% (nivolumab plus ipilimumab), 13% (nivolumab), and 10% (chemotherapy), with a HR for OS for nivolumab plus ipilimumab of 0.64 (95% CI 0.51-0.81) suggesting durable benefit in a subset of patients (94).

The CheckMate 9LA trial explored the role of first-line nivolumab plus ipilimumab in combination with two cycles of chemotherapy (74). In this trial, patients were randomized to either nivolumab plus ipilimumab plus two cycles of platinum doublet chemotherapy or chemotherapy alone. Interim analysis showed increased OS in the experimental group (median 14.1 months vs 10.7 months, 95% CI 9.5 -12.6, HR 0.66, 95% CI 0.55-0.80). Notably, any-grade treatment-related adverse events occurred in 30% of the experimental group and in 18% of the control group. On the basis of this trial, fit patients who have significant disease burden are suggested to benefit from upfront chemotherapy in combination with dual checkpoint blockade. Importantly this trial did not show an early decrease in PFS compared to other trials. Furthermore, with only two cycles of chemotherapy, there is potential to avoid long-term chemotherapy-related toxicity, thus presenting a unique treatment option. This trial established the role of dual-checkpoint blockade as a reasonable first-line treatment option in metastatic NSCLC. The two-year update presented at ASCO 2021 reported that patients who received the experimental combination treatment derived OS benefit compared to the control arm, with OS of 15.8 months vs 11.0 months (HR 0.72, 95% CI 0.61-0.86), PFS benefit of 6.7 months vs 5.3 months (HR 0.67, 95% CI 0.56-0.79), and improved ORR (38% vs 25%), with similar benefits being seen across varying PD-L1 expression levels (75). Importantly, a *post hoc* analysis of the 14% of patients with brain metastases showed that this chemo-immunotherapy combination was associated with benefit this patient in this subset of patients (95).

A similar approach of first-line dual checkpoint blockade in metastatic NSCLC was explored in the phase 3 POSEIDON trial and was presented at WCLC in 2021. In this trial, durvalumab (anti-PD-L1 mAb) and tremelimumab (anti-CTLA4 mAb) plus chemotherapy was shown to be superior to chemotherapy alone with a PFS benefit (6.2 months vs 4.8 months, HR 0.72, 95% CI 0.60-0.86, p = 0.00031) as well as an OS benefit (14.0 months vs 11.7 months, 95% CI 11.7-16.1, p = 0.00304). Notably this trial reported a 51.8% grade ¾ treatment-related adverse effect rate with 15.5% of patients requiring discontinuation of study treatments. Interestingly, the addition of tremelimumab was not associated with a large difference in treatment discontinuation rates compared to the durvalumab plus chemotherapy arm (14.1%) (96). This finding in particular supports the notion that dual checkpoint blockade with PD-1/PD-L1 and CTLA-4 blockade may not necessarily be more toxic than single agent PD-1/PD-L1 blockade and could represent yet another first-line option in the treatment of metastatic NSCLC.

#### Checkpoint inhibitors combined with angiogenesis inhibitors

The Impower150 trial randomized patients with nonsquamous metastatic NSCLC to receive either atezolizumab plus carboplatin and paclitaxel (ACP), or bevacizumab plus carboplatin and paclitaxel (BCP), or atezolizumab plus bevacizumab plus carboplatin and paclitaxel (ABCP), followed by atezolizumab or bevacizumab maintenance, or both. This trial found improved PFS (8.3 months vs 6.8 months, HR 0.62, 95% CI 0.52-0.74, p<0.001) and improved OS (19.2 months vs 14.7 months, HR 0.78, 95% CI, 0.64-0.96, p=0.02) with the addition of atezolizumab to bevacizumab plus chemotherapy regardless of PD-L1 expression and *EGFR/ALK* status (38).

Many immunotherapy trials have historically excluded patients with untreated brain metastases and thus the efficacy of immunotherapy for CNS control has not been fully elucidated. Cemiplimab monotherapy was examined in patients with PD-L1 ≥ 50% in a subgroup analysis of the EMPOWER-Lung 1 trial. This trial showed improved PFS (10.4 months vs 5.3 months, HR 0.45, 0.22-0.92, p=0.0231) and OS (18.7 months vs 11.7 months, HR 0.17, 0.04-0.76, p=0.0091) compared to chemotherapy alone in patients with treated, clinically stable brain metastases (79). To further study CNS disease, the ATEZO-BRAIN study was a phase II trial of atezolizumab with carboplatin and pemetrexed in patient with metastatic nonsquamous NSCLC and untreated brain metastases. This study evaluated 40 patients and noted a median PFS of 8.9 months and a median OS of 13.6 months. Importantly 36 of 40 patients had concordant response between CNS disease and systemic disease suggesting that immunotherapy with chemotherapy can be utilized in patients with brain metastases without necessitation of local CNS therapy (97). Yet another sub group analysis evaluating brain metastases in the CheckMate 9LA trial showed that immunotherapy led to improved survival compared to chemotherapy alone and showed similar 2-year OS rates in patients who received nivolumab, ipilimumab, and chemotherapy with brain metastases and those without (25% vs 39%) (98). These data together strongly suggest the utility of immunotherapy alone or in combination with chemotherapy in patients with CNS involvement.

#### Oncogene driven NSCLC

Despite successes of ICIs in patients with wild type NSCLC, their efficacy in *EGFR* and *ALK* mutant NSCLC is disappointing. Their low efficacy was first observed in second line studies where single agent PD-1 inhibitors had no benefit over docetaxel in subgroup analysis. Real world data from the ImmunoTarget Global registry also shows that patients without actionable mutations and smokers are more likely to respond to immunotherapy. Specifically, evaluation of this registry showed that patients with *KRAS*, *BRAF*, and *MET* alterations responded more favorably than patients with *EGFR*, *ALK*, or *RET* alterations (99, 100). A phase 2 trial of pembrolizumab in TKI naïve, PD-L1-positive *EGFR*-mutant advanced NSCLC was stopped due to poor efficacy, providing clinical data discouraging the use of immunotherapy in this patient population, despite otherwise encouraging PD-L1 levels (47). Translational studies have demonstrated that *EGFR-*mutant NSCLC tend to have an immune excluded phenotype where anti-tumor T cells are unable to traffic into the tumor microenvironment (101, 102). Efforts to combine ICI inhibitors and targeted TKIs were disastrous with significant increases in severe toxicity such as pneumonitis and hepatitis (103, 104). This area remains an unmet need and is topic of active investigation and highlights the necessity of genomic profiling in order to guide treatment choices.

### Biomarkers in immuno-oncology

#### PD-L1 tumor proportion score

The most commonly reported and used biomarker for immunotherapy is PD-L1 expression. There are various PD-L1 assays that are currently being utilized. The companion diagnostic for nivolumab utilizes an assay developed by Dako (5H1 or 28-8 assay), whereas pembrolizumab uses the Dako 22C3 assay. Many of the IMpower trials investigating atezolizumab utilized the Ventana SP263 assay and assessed PD-L1 expression on both tumor cells and immune cells (105). Higher PD-L1 expression is predictive of response, particularly for ICI monotherapy as demonstrated by many of the first line studies discussed. The Blueprint PD-L1 IHC Assay Comparison Project evaluated comparability of the different IHC assays and found that the Ventana SP263 assay stained fewer tumor cells. Furthermore, the study noted that 37% of tumors received different classifications depending on which IHC assay was being utilized (106). In addition to the Blueprint Project, there is additional data available to suggest that PD-L1 expression is heterogenous within tumors and thus, random biopsy may not be indicative of a true immunosuppressed state (107–110). These inherent flaws in IHC evaluation of PD-L1 expression may, at least in part, explain the variability in response rates seen in tumors with similar PD-L1 expression. The role of PD-L1 expression as a biomarker in early stage NSCLC is controversial. A study reported in 2015 showed that PD-L1 expression was a favorable prognostic indicator (111), however other studies suggested no prognostic value (112) questioning the utility of this biomarker especially in early stage disease.

#### Tumor Mutational Burden (TMB)

Tumor mutational burden (TMB) has been investigated as a potential biomarker for immunotherapy response however results have been conflicting. The initial interest in TMB stemmed from a study conducted by Rizvi et al, who conducted whole-exome sequencing on NSCLC specimens that had been treated with pembrolizumab. Investigators found improved objective response, durable clinical benefit, and PFS in tumors which had higher nonsynonymous mutation burdens. Furthermore, higher mutation burden was associated with higher neoantigen burden and DNA repair pathway mutations (113).

In the CheckMate-026 trial, TMB analysis using whole exome sequencing (WES) was conducted. This analysis showed that patients with high TMB and treated with nivolumab had higher ORR (47% vs 28%) and median PFS (9.7 months vs 5.8 months, HR 0.62, 95% CI 0.38-1.00) compared to those treated with chemotherapy. Notably, there was no correlation seen between TMB and PD-L1 expression. Lastly, ORR was increased when selecting for patients with both high TMB and PD-L1≥50%, however increased TMB level did not translate into better OS (77).

The CheckMate-227 trial demonstrated PFS benefit with combination nivolumab and ipilimumab compared to chemotherapy in first-line treatment of NSCLC with high TMB (≥10 mutations per megabase) (114). Further exploratory analyses combining both PD-L1 expression and TMB failed to reveal a subgroup that had an increased benefit from combination nivolumab and ipilimumab. Given these data, the utility of TMB as a biomarker has been brought into question.

A novel assay developed by Foundation Medicine allowed assessment of blood TMB (bTMB), and was retrospectively studied using samples from the phase 2 POPLAR and phase 3 OAK trials to identify predictive thresholds. In this study, patients with bTMB ≥ 16 mutations (16 mut/Mb) who were treated with atezolizumab had improved PFS as compared to patients with the same bTMB but treated with docetaxel (HR 0.65, 95% CI 0.47-0.92, p=0.013) (115). This assay is currently being studied in two prospective trials: Blood First Assay Screening Trial (BFAST) and in the Blood First-Line Ready Screening Trial (B-F1RST). Interim results of the B-F1RST trial showed that bTMB has value as a predictive biomarker as it relates to PFS (PFS for bTMB high was 9.5 months vs 2.8 months for bTMB low, HR 0.49, 90% CI, 0.23-1.04, p=0.11) and ORR (ORR for bTMB high was 36.4% versus 6.4% for bTMB low, OR 8.38, 90% CI 2.02-34.79, p = 0.02) (116). Final efficacy results showed an ORR of 17%, median PFS of 4.1 months, and median OS of 14.8 months. In bTMB of ≥ 16 mutations compared to <16, median OS was 23.9 months vs 13.4 months (117).

The MYSTIC trial was a lesson in patient selection. In this trial, patients with metastatic NSCLC without *EGFR* or *ALK* alterations were stratified based on tumor PD-L1 expression of ≥25% or ≤25% and histological classification. In this trial, 1118 patients were randomized to either durvalumab, durvalumab plus tremelimumab (anti-CTLA-4 mAb), or platinum-doublet chemotherapy and did not meet its primary endpoint of improved OS in the experimental arms (118, 119). PD-L1 expression was determined based on the VENTANA PD-L1 (SP262) assay. Perhaps more interesting is that an exploratory analysis showed that patients who had a high bTMB (≥20) and were treated with durvalumab and tremelimumab had OS of 21.9 months compared to 10 months in patients treated with chemotherapy (HR 0.49, 95% CI, 0.32-0.74) (119, 120).

The cancer gene panel (CGP), NCC-GP150, that serves as an estimation of blood TMB, was found to be a potentially useful tool in the treatment of NSCLC. Published in JAMA Oncology in 2019, Wang et al, demonstrated in two independent cohorts of patients, that the NCC-GP150 correlated to tissue TMB as determined by WES. bTMB of ≥ 6 (units) was associated with better PFS (HR 0.39; 95% CI 0.18-0.84, p = 0.01). ORR was also higher in tumors with bTMB ≥6 at 39.3% (95% CI 23.9%-56.5%) compared to 9.1% in tumors with bTMB <6 (95% CI, 1.6%-25.9%) (121).

Somewhat unexpectedly, higher TMB has been associated with earlier stages of disease. In a study of 197 samples, TMB was higher in stage I and II than in stage III and IV tumors (67% vs 47.5%, p=0.01) (122). The clinical value of this finding is not clear, but it highlights the fact that broad statements about TMB cannot be made without the context of related variables such as stage. Despite the confusing and conflicting results discussed, there is likely value of TMB as biomarker. Recently, pembrolizumab received regulatory approval for management of TMB-High solids tumors (≥10 mut/Mb) based on a biomarker directed analysis of 10 cohorts of commonly occurring solid tumors, but which did not include NSCLC (123). Given that the current use of ICIs in NSCLC is mainly driven by PD-L1 expression, which is supported by clinical trial data, the role of TMB as a biomarker for NSCLC remains yet to be determined.

### Future directions for immunotherapy in NSCLC

The overall response rate of immunotherapy in NSCLC is about 20-30%, which means that a significant proportion of patients do not have meaningful responses. Why patients, who on the surface have similar tumors, can have very different responses to immunotherapy is a complicated question, one without clear answers. In some part, lack of response to immunotherapy can be blamed on the lack of activated immune cells within the tumor microenvironment (TME).

**Figure 1:**
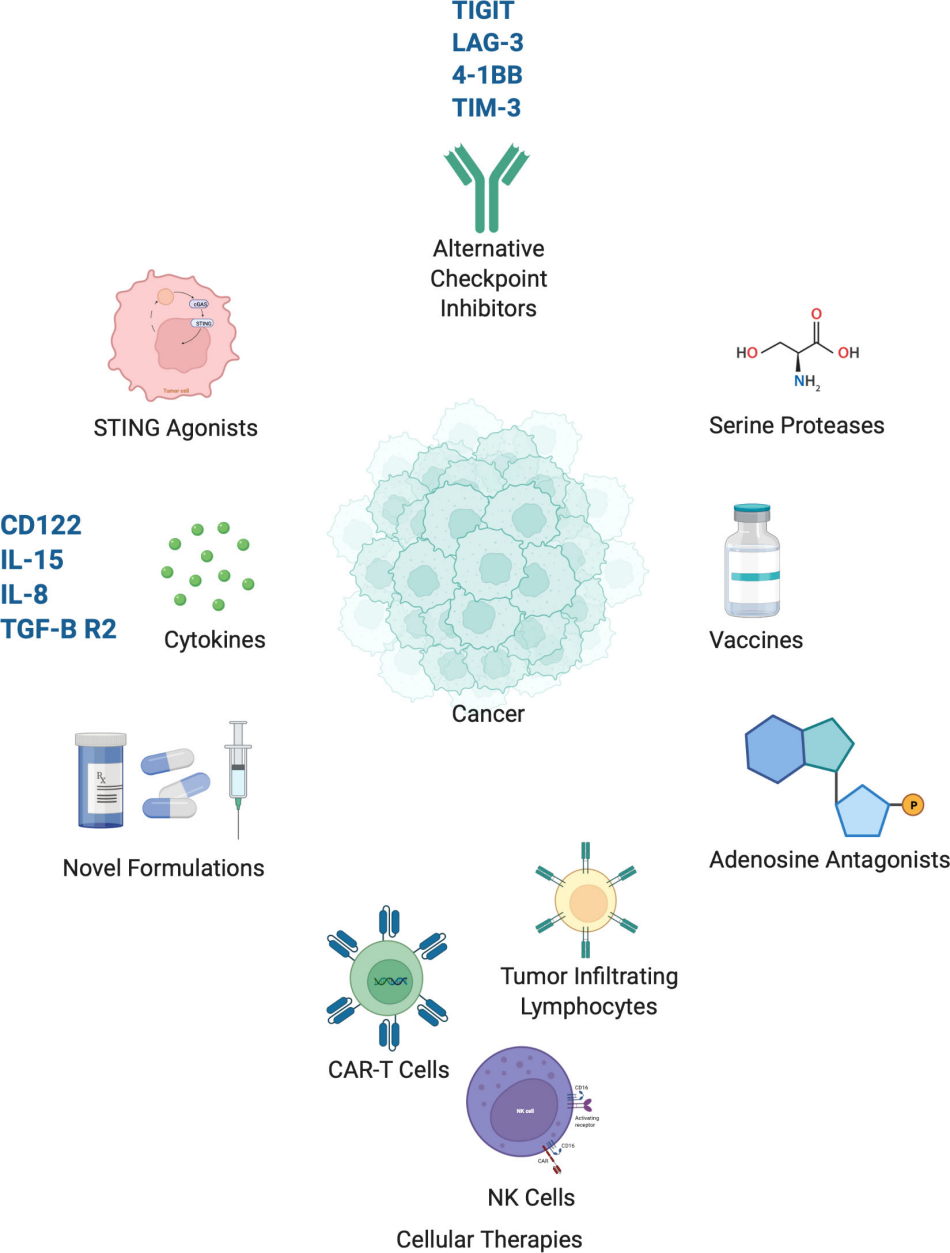
Select emerging immunotherapies currently in clinical development. Created with BioRender.com.

The future of immuno-oncology for the treatment of lung cancer lies in two approaches – the first is by improving the efficacy of treatment by combining it with modalities that enhance the immune response or through novel therapies (**Table 2**). The second is by improving patient selection by further developing biomarkers that can identify patients most likely to benefit from immunotherapy and integrating new technology into this process of patient selection. Here we discuss several novel treatment approaches which are in development (**Figure 1**).

**Table 2:** Select clinical trials currently underway which utilize investigational agents in combination with checkpoint inhibitors.

Study	Phase	Indication	Investigational Agent	Therapy
PIVOT (NCT02983045)	1/2	MEL, RCC, NSCLC, TNBC, UC	NKTR-214 (CD122-biased agonist)	NKTR-214 in combination with nivolumab (anti-PD-1 ab)
NCT02403193	1/2	NSCLC	NIR178 (PBF-509) (A2AR antagonist)	Single agent NIR178 or in combination with PDR001 (anti-PD-1 ab)
NCT03388632	1	Advanced solid malignancies	Subcutaneous rhIL-15	IL-15 with nivolumab (anti-PD-1 ab) and/or ipilimumab (anti-CTLA-4 ab)
NCT03400332	1b/2	Advanced solid malignancies	BMS-986253 (anti-IL-8 ab)	BMS-986253 in combination with nivolumab (anti-PD-1 ab)
NCT03005782	1	Advanced malignancies	REGN3767 (anti-LAG-3 ab)	Single agent REGN3767 or in combination with cemiplimab (anti-PD1 ab)
NCT03099109	1	Advanced solid malignancies	LY3321367 (anti-TIM3 ab)	Single agent LY3321367 or in combination with LY3300054 (anti-PD-L1 ab)
NCT03164772	1/2	NSCLC	BI 1361849 (mRNA vaccine)	BI 1361849 with durvalumab (anti-PD-L1 ab) and/or tremelimumab (anti-CTLA-4 ab)

#### Other immune checkpoints

Tiragolumab, monoclonal antibody against the inhibitory checkpoint TIGIT, in combination with atezolizumab has been given breakthrough therapy designation by the FDA for the first-line treatment of PD-L1 positive metastatic NSCLC. This designation was granted on the basis of the CITYSCAPE trial. In this phase II trial, the ORR was 37.3% in the combination arm, compared to 20.6% in the atezolizumab only arm. Furthermore, PFS was 5.6 vs 3.9 months in the combination arm and atezolizumab arm, respectively (124). The phase 3, SKYSCRAPER-01 trial is currently underway, however interim results released in May 2022 showed that the trial did not meet its co-primary end point of PFS (NCT04294810, Roche News Release 5 11 2022).

Lymphocyte activation gene 3 (LAG-3) has emerged as another co-inhibitory checkpoint receptor in the regulation of T cells and evidence suggests that dual expression of LAG-3 and PD-1 is associated with T cell anergy and subsequent immunosuppression within the TME. Preclinical data shows that LAG-3 inhibition alongside PD-1 inhibition can have strong anti-tumor activity (125). Study of REGN3767 (an anti-LAG-3 ab) as monotherapy and in combination with cemiplimab (anti-PD1 ab) is currently underway (126, 127). Importantly, there appears to further ligands to LAG-3, specifically fibrinogen-like protein 1 (FGL1) (128), which may have implications for further drug discovery.

4-1BB/CD137 is a costimulatory receptor and is part of the TNF receptor superfamily. Engagement of this receptor enhances T and NK cell activity and has been shown to produce anti-tumor effects. Utolimumab (PF-05082566), a 4-1BB/CD137 agonist was studied in a phase 1 clinical trial in patients with advanced malignancies as a single agent (129) and in combination with pembrolizumab (130) and was shown to be well tolerated and had positive preliminary clinical activity. Utolimumab is currently in trials in combination with an OX40 agonist, another co-stimulatory checkpoint, (NCT02315066) and in combination with avelumab (anti-PD-L1 mAb) (NCT02554812).

Data from a phase 1 trial utilizing an anti-T-cell immunoglobulin domain and mucin domain–containing molecule-3 (TIM-3) (LY3321367) antibody as a single agent or in combination with LY3300054 (anti-PD-L1 mAb) was presented at the 2019 ASCO-SITC meeting and showed positive safety results (131). Such combination therapies have great potential to improve responses in patients already responding to immunotherapy and induce responses in those whose tumors are inherently less responsive.

#### Cytokines

Combination therapy of cytokines with immunotherapy has been the focus of much attention for many years as it attempts to solve a central problem in tumor immune-escape, namely the immunosuppressive quality of the TME. Herein we discuss several important compounds and treatment strategies currently under development.

The PIVOT trial (NCT02983045) is a phase 1/2 study of combination therapy with NKTR-214 (bempegaldesleukin) and nivolumab in advanced malignancies including NSCLC (132, 133). NKTR-214 is a CD122-biased agonist (CD122 is also known as the interleukin-2 receptor beta subunit) that was developed on the basis of prior experience with IL-2 in the treatment of malignancy. This compound functions to increase proliferation of CD8+ effector and natural killer (NK) cells within the TME (134). As discussed previously, there is a direct correlation between TIL concentration and response to immunotherapy (135–137). Encouraging ORR and DCR responses have been seen (132). The currently underway phase I/II PROPEL trial (NCT03138889) includes first-line NSCLC patients for treatment with bempegaldesleukin and pembrolizumab with or without chemotherapy.

IL-15 has long been studied for its potential anti-tumor effects for its T and NK cell stimulatory effects (without stimulation of Tregs). A phase 1 dose-escalation trial utilized subcutaneous recombinant IL-15 (rhIL-15) to achieve much higher doses than possible when given intravenously. Nineteen total patients were treated, including 6 with NSCLC (138). While this study did not detect objective responses, a clinical trial is underway (NCT0338863) that is testing rhIL-15 with nivolumab, with ipilimumab, and with both ipilimumab and nivolumab in advanced solid malignancies. ALT-803 is an IL-15/IL-15Rα complex fused to an IgG1 Fc, referred to as an IL-15 superagonist. In this compound, the IL-15 is mutated to increase agonism of IL-2 and 15βγ receptors. A phase 1b trial of ALT-803 in combination with nivolumab in advanced NSCLC showed adequate safety and encouraging clinical activity with an ORR of 29% (139).

IL-8 has been known to have protumoral effects, through multiple proposed mechanisms (140). Interestingly, Sanmamed et al, showed that low IL-8 levels in melanoma and NSCLC are associated with better response to immunotherapy (141). A phase 1 trial of BMS-986253, an anti-IL-8 antibody, has been completed and showed that monotherapy is well tolerated (142). NCT03400332 is a trial that is currently underway in which BMS-986253 is being evaluated in combination with nivolumab (143).

M7824, developed by EMD Serono, is a unique bifunctional protein which contains anti-PD-L1 1gG1 mAb that is fused with 2 extracellular domains of TGF-B receptor II to form a so called “TGF-B trap.” This compound attempts to circumvent a classic problem with ICB, namely tumor immunosuppression. This molecule has been shown to impede TGF-B related signaling in the TME and, in animal models, to be more effective than either TGF-B inhibition or PD-L1 inhibition (144). A second line trial of 80 patients with NSCLC showed impressive efficacy with ORR of 85.7% in patients with tumors with PD-L1 ≥ 80% and 36% in PD-L1 ≥ 1% at the recommended phase 2 dose (145).

#### STING agonists

In an attempt to counteract this dearth of immune cells in the TME, several therapeutic avenues are being evaluated. Intratumoral injection of a STING (stimulator of interferon genes) agonist (cGAMP) has been shown in non-inflamed lung cancer models to increase intra-tumoral CD8+ T cells, increase PD-1, PD-L1, IFN concentrations, and synergistically suppressed tumor growth in cells otherwise resistant to immune checkpoint inhibitors (ICIs) when combined with anti-PD-L1 ab (146). Preliminary results from a phase 1 trial of MK-1454 (a STING agonist) showed encouraging safety and efficacy when combined with pembrolizumab (147). Another example of this approach is with sitravatinib, a tyrosine kinase inhibitor which targets TAM and VEGFR2, reducing immunosuppressive MDSCs and Tregs but increasing M1/M2-polarized macrophages and thereby overcoming the immunosuppressive TME. A phase 2 trial was presented at ESMO 2021 which showed favorable survival data when used in combination with nivolumab in the second and third line treatment of advanced NSCLC (148). A plethora of approaches are currently underway which utilize intratumoral administration of various forms of immunotherapy, including gene therapies, cellular therapies, and immunostimulatory compounds are currently being evaluated (149).

#### Adenosine antagonists

Adenosine is a unique compound in regard to its role in tumorigenesis. The TME is a hypoxic environment which stimulates the release of ATP which subsequently activates an inflammatory response (150). In order to counterbalance this effect, ATP is converted to adenosine, which produces a profound anti-inflammatory response *via* inhibition of many cells including mast cells, endothelial cells, macrophages, neutrophils, NK cells, dendritic cells, and lymphocytes (151); adenosine has a potent effect on inducing T cell anergy and promotes the differentiation of CD4 cells into Foxp3^+^ Tregs (151, 152). Furthermore, hypoxia within the TME results in generation of HIF1α (hypoxia inducible factor 1α) which further induces adenosine receptor A2BR (A2AR and A2BR primarily have immunosuppressive qualities) (153, 154). Overexpression of these molecules has been associated with metastatic disease and worse clinical outcomes in various tumor types (155, 156). As mentioned previously, it is theorized that this immunosuppressed state within the TME results in poor responses to immunotherapy. CPI-444, developed by Corvus Pharmaceuticals, is an orally available A2AR antagonist which was shown in a preclinical mouse model to work synergistically with anti-PD-L1 therapy or anti-CTLA therapy and produce complete responses in 90% of mice; furthermore, this therapy was shown to induce a memory response in treated mice (157). PBF-509 (NIR178) is another A2AR antagonist that has shown promising results in a murine model by restoring TIL activity (158). A phase 1/2 trial in advanced NSCLC suggests adequate safety as well as clinical benefit (159). This study also plans to treat patients with PBF-509 in combination with PDR001 (anti PD-1 mAb). This unique direct modulation of the immune response by adenosine allows for improvement of the anti-tumor immune response using well-studied checkpoint inhibitors (PD-1, PD-L1, CTLA4 inhibitors) in combination with metabolically active agents. As adenosinergic molecules are further developed, we expect to see them more widely tested in combination with currently approved therapies.

#### Cellular therapies

Cellular therapies are another type of therapeutic approach that is being evaluated in the treatment of NSCLC. There are several different kinds of cellular therapies, the most well studied in solid malignancies are chimeric antigen receptor-modified T (CAR-T) therapy, adoptive cell therapy (ACT) and cancer vaccine therapy. A phase 1 trial of CAR-T cell technology directed to EGFR in NSCLC was published in 2016 and showed adequate safety (160). There have been many studies that have suggested the use of various targets in NSCLC for further CAR-T development such as EphA2, EGFR, HER2, MSLN, MUC1, ROR1, PD-L1, and PSCA (161–163). A form of T cell therapy developed by Adaptimmune is ADP-A2M10 which utilizes affinity-enhanced autologous T cells directed against the MAGE-A10^c796^ tumor antigen. Initial safety data from phase 1 clinical trials, presented at ASCO in 2018,were promising (164). A phase 1 trial of BI 1361849, an mRNA vaccine which contains several NSCLC antigens (MUC1, survivin, NY-ESO-1, 5T4, MAGE-C2, and MAGE-C1) is currently underway in combination with durvalumab (anti-PD-L1 ab) or in combination with both durvalumab and tremelimumab (anti-CTLA-4 mAb) (165). Tumor infiltrating lymphocyte (TIL) therapy as a form of adoptive cellular therapy has shown encouraging results in 20 patients with advanced NSCLC (NCT03215810). In this trial, autologous TILs were surgically harvested from patient tumors and were expanded ex vivo. After lymphodepleting and infusion of TILs, the cells were expanded *in vivo* utilizing IL-2 (166). This approach garners much attention as it an example of personalized medicine at its best and has the potential to induce durable immune responses. In fact, the trial mentioned had 2 complete responders (166). Another form of cellular therapy, NK cells, and in this case, NK cells manufactured from induced pluripotent stem cells, have been shown to increase T cell recruitment within tumors and synergize with anti-PD-1 antibodies in preclinical models (167). This form of therapy has entered clinical trials (NCT05069935).

Vaccination against PD-L1 is an interesting approach to decrease immunosuppression within the tumor microenvironment that deserves attention. IO102-IO103 is a cancer vaccine targeting IDO and PD-L1 that has shown encouraging safety and efficacy data in metastatic melanoma (168) and is currently in Phase 2 clinical trials in combination with pembrolizumab in patients with NSCLC (NCT05077709).

#### Novel formulations of immune checkpoint inhibitors

While still early in development, orally available small molecule PD-L1 inhibitors, such as CA-170, have been developed which show preclinical efficacy and hold promise of easy administration and improved pharmacokinetic properties (169). Probody therapeutics (Pb-tx) are a form of antibody prodrugs which are activated by proteases. The mechanistic underpinning of this approach is that antibody can be better delivered to the tumor microenvironment as is it reliant on protease activation. These therapies have preclinically been shown to have similar activity as their traditional anti-PD-1 and anti-PD-L1 mAbs, however with less systemic toxicity (170). CX-072, an anti-PD-L1 Pb-Tx is currently in the clinic and is associated with good tolerability as well as antitumor efficacy (171). De-fucosylated (or non-fucosylated) antibodies have long been studied as means to improve antibody efficacy. De-fucosylated anti-PD-L1 antibodies have been shown to have increased antibody dependent cellular cytotoxicity *via* improved FcγRIIIa binding (172). We anticipate further study and optimization of these novel formulations of immune checkpoint inhibitors and are hopeful that they will improve the effectiveness of these agents.

#### Other emerging therapies

Serine proteases have also been suggested as a potential avenue to increase antigen presentation. Serine proteases within the TME have been shown to increase HLA-1 expression of peptides by lung cancer cells (173). While this approach is still in its infancy, we expect this approach and other similar ones to be further studied and enter the clinics soon.

#### Emerging biomarkers: Gene expression signatures

Data suggest that patients with advanced NSCLC whose tumors have high PD-L1 expression but low cytotoxic T (CD8+ T) cell tumor infiltrating lymphocytes (TILs) have a poor prognosis with median OS of 3.7 months compared to 8 months for patients with tumors not exhibiting this fatal combination (p=0.02). In this study, investigators identified a CD8A/CD274 gene signature (evaluation of mRNA by RNA sequencing) that was able to predict tumors that would respond to ICB (174). These results are in concordance with prior studies which demonstrate that in NSCLC, increased TIL concentrations are associated with better prognoses (135, 136, 175, 176). TIL concentrations have been evaluated as a potential predictive biomarker (137, 177), however the previously stated findings support the notion that TIL concentration in combination with PD-L1 expression can be used as a predictive biomarker for response to immunotherapy (174).

An exploratory analysis of the RATIONALE-307 was presented at WCLC 2021. In this biomarker evaluation, investigators found an association between tumor inflammation signature (TIS, a 18-gene panel representing inflammatory genes) and benefit of PD-1 inhibition with tislelizumab (anti-PD-1 mAb) in combination with chemotherapy compared to chemotherapy alone in the first-line treatment of advanced squamous NSCLC (178). These data suggest the utility of TIS as a biomarker more so than even PD-L1 and tTMB.

Given the potential pitfalls associated with the above-mentioned biomarkers, continued study is currently underway to identify more efficient and more accurate biomarkers, some of which are discussed here. Interferon gamma (IFN-γ) expression, in recent studies, has been evaluated as a potential biomarker that is suggested to predict response to immunotherapy (179–181). The intestinal microbiome has been shown to influence the efficacy of immunotherapy and has subsequently been suggested as a potential predictive biomarker for response to checkpoint blockade (182–184). Janus Kinase (*JAK) 1* and *2* mutations as well as *beta-2-microglobulin* (*B2M*) aberrations and T cell immunoglobulin mucin-3 (TIM-3) upregulation have also been suggested as potential biomarkers that are currently undergoing study but have yet to be validated (185, 186).

#### Radiomics as a biomarker

Patient selection can be improved upon by utilizing improved biomarkers as well as artificial intelligence (AI) based approaches to treatment of NSCLC. As previously discussed in this review, high concentrations of TILs are associated with improved prognoses and better responses to immunotherapy. A recent study by Corredor et al, was able to predict the likelihood of recurrence in stage I and II NSCLC by evaluating computer-extracted spatial TIL (SpaTIL) features from digital images of tumors (187). This concept, if broadened, may help identify patients who are most likely to respond to immunotherapy and may guide adjunctive therapy selection for patients who are deemed less likely to respond. Developments in deep learning and radiomics may also aide in prognosticating patients based on radiographic appearance (188). In a study published by Coudray et al. in 2018, investigators were able to train a deep convolutional neural network (CNN) on pathology images and was able to predict histology as well as identify 6 commonly mutated genes (*STK11, EGFR, FAT1, SETBP1, KRAS and TP53*) (189). Radiomics is quickly emerging as a field with great potential. Radiomic based approaches have been developed to predict the benefit of adjuvant chemotherapy in early-stage NSCLC (190). These approaches have been shown to predict survival and response to immunotherapy in advanced NSCLC (191). Quantitative vessel tortuosity (QVT), a set of features evaluating the quantity and quality of vessels as measured on CT scans, is also gaining popularity as a potential biomarker (192). As this technology is further developed, we can envision its applications into producing biomarkers for response and improving patient selection for immunotherapy.

## Discussion

In this paper, we have reviewed the development of immunotherapy and its initial application to NSCLC. We have also reviewed many of the landmark clinical trials that have changed practice and led to FDA approvals. Biomarkers play a large role in selecting patients most likely to respond to immunotherapy, but thus far, efforts remain imperfect as accuracy and efficiency are being sought after. Continued research into novel biomarkers will be paramount to the immunotherapy field. Despite all of these momentous advancements in the treatment of an otherwise dismal disease, there is still much potential for improvement. Several strategies have been employed to improve responses to immunotherapy, such as improving antigen presentation, combinations with cytokines, and cellular therapies. Lastly, we have reviewed up and coming science that may aide in improving patient selection and prediction of response to immunotherapy.

## Author contributions

SP, ES, CG, SL, and VV contributed to writing of this manuscript. All authors contributed to the article and approved the submitted version.

## Conflict of interest

The authors declare that the research was conducted in the absence of any commercial or financial relationships that could be construed as a potential conflict of interest.

## Publisher’s note

All claims expressed in this article are solely those of the authors and do not necessarily represent those of their affiliated organizations, or those of the publisher, the editors and the reviewers. Any product that may be evaluated in this article, or claim that may be made by its manufacturer, is not guaranteed or endorsed by the publisher.
